# Integrated transcriptomics uncovers an enhanced association between the prion protein gene expression and vesicle dynamics signatures in glioblastomas

**DOI:** 10.1186/s12885-024-11914-6

**Published:** 2024-02-13

**Authors:** Jacqueline Marcia Boccacino, Rafael dos Santos Peixoto, Camila Felix de Lima Fernandes, Giovanni Cangiano, Paula Rodrigues Sola, Bárbara Paranhos Coelho, Mariana Brandão Prado, Maria Isabel Melo-Escobar, Breno Pereira de Sousa, Shamini Ayyadhury, Gary D. Bader, Sueli Mieko Oba Shinjo, Suely Kazue Nagahashi Marie, Edroaldo Lummertz da Rocha, Marilene Hohmuth Lopes

**Affiliations:** 1 Department of Cell and Developmental Biology, Institute of Biomedical Sciences, University of São Paulo, Av. Prof. Lineu Prestes, 1524 room 431, Sao Paulo, 05508000 Brazil; 2 Department of Automation and Systems, Technological Center, Federal University of Santa Catarina, Florianópolis, Santa Catarina Brazil; 3 Cellular and Molecular Biology Laboratory (LIM 15), Department of Neurology, Faculdade de Medicina (FMUSP), University of Sao Paulo, Sao Paulo, Brazil; 4 Princess Margaret Cancer Centre, University Health Network, Toronto, Ontario Canada; 5 The Donnelly Centre, University of Toronto, Toronto, Ontario Canada; 6 Department of Molecular Genetics, University of Toronto, Toronto, Ontario Canada; 7 Department of Computer Science, University of Toronto, Toronto, Ontario Canada; 8 Department of Microbiology, Immunology, and Parasitology, Biological Sciences Center, Federal University of Santa Catarina, Florianópolis, Santa Catarina 88040-900 Brazil

**Keywords:** Glioblastoma, Transcriptomics, Prion protein, Intracellular trafficking, Vesicle dynamics

## Abstract

**Background:**

Glioblastoma (GBM) is an aggressive brain tumor that exhibits resistance to current treatment, making the identification of novel therapeutic targets essential. In this context, cellular prion protein (PrP^C^) stands out as a potential candidate for new therapies. Encoded by the *PRNP* gene, PrP^C^ can present increased expression levels in GBM, impacting cell proliferation, growth, migration, invasion and stemness. Nevertheless, the exact molecular mechanisms through which *PRNP*/PrP^C^ modulates key aspects of GBM biology remain elusive.

**Methods:**

To elucidate the implications of *PRNP*/PrP^C^ in the biology of this cancer, we analyzed publicly available RNA sequencing (RNA-seq) data of patient-derived GBMs from four independent studies. First, we ranked samples profiled by bulk RNA-seq as *PRNP*^high^ and *PRNP*^low^ and compared their transcriptomic landscape. Then, we analyzed *PRNP*^+^ and *PRNP*^-^ GBM cells profiled by single-cell RNA-seq to further understand the molecular context within which *PRNP*/PrP^C^ might function in this tumor. We explored an additional proteomics dataset, applying similar comparative approaches, to corroborate our findings.

**Results:**

Functional profiling revealed that vesicular dynamics signatures are strongly correlated with *PRNP*/PrP^C^ levels in GBM. We found a panel of 73 genes, enriched in vesicle-related pathways, whose expression levels are increased in *PRNP*^high^/*PRNP*^+^ cells across all RNA-seq datasets. Vesicle-associated genes, *ANXA1*, *RAB31*, *DSTN* and *SYPL1,* were found to be upregulated *in vitro* in an in-house collection of patient-derived GBM. Moreover, proteome analysis of patient-derived samples reinforces the findings of enhanced vesicle biogenesis, processing and trafficking in *PRNP*^high^/*PRNP*^+^ GBM cells.

**Conclusions:**

Together, our findings shed light on a novel role for PrP^C^ as a potential modulator of vesicle biology in GBM, which is pivotal for intercellular communication and cancer maintenance. We also introduce GBMdiscovery, a novel user-friendly tool that allows the investigation of specific genes in GBM biology.

**Supplementary Information:**

The online version contains supplementary material available at 10.1186/s12885-024-11914-6.

## Introduction

Glioblastoma (GBM) is an aggressive central nervous system tumor classified as a grade IV glioma by the World Health Organization (WHO) [1]. At the molecular level, the mutation status of isocitrate dehydrogenase (IDH) is an important diagnostic and prognostic biomarker. Survival expectancy for patients bearing IDH wild-type (IDHwt) GBM is dismal, and this type of cancer is more aggressive than the IDH-mutant counterpart. Given the differences between IDHwt and IDH-mutant tumors, a new classification defining GBM exclusively as IDHwt cancer has been proposed [2]. Transcriptionally, GBM is stratified into three molecular subtypes: proneural, classical, and mesenchymal [3]. Recently, cells resembling different molecular subtypes and spanning distinct genetic programs were shown to exist within a single tumor, highlighting the heterogeneous nature of this cancer [4]. In this context, GBM bears a subpopulation of glioma stem-like cells (GSCs) associated with its heterogeneity, aggressiveness, and therapy resistance [5].

In the past 20 years, standard treatment delivered to individuals with GBM has been surgical resection followed by radiotherapy and chemotherapy with temozolomide, providing patients with a median overall survival of 14-20 months [6–8]. Several aspects are proposed to be associated with GBM malignancy. Low therapy effectiveness is partially explained by resistance mechanisms to both radio and chemotherapy [9], and surgical resection is hindered by the invasive nature of GBM [7]. GSCs may play a role in therapy resistance by releasing extracellular vesicles (EVs), which carry bioactive molecules (protein, RNA, DNA, sugar, and lipids) able to manipulate the tumor microenvironment to support tumor growth and progression [10, 11].

Research efforts have focused on identifying novel potential molecular targets to be exploited in GBM therapeutics and to give patients better life expectancy and quality. Increasing evidence has demonstrated that the cellular prion protein (PrP^C^) plays a key role in GBM biology. PrP^C^ is a glycosylphosphatidylinositol-anchored cell surface glycoprotein that faces the extracellular space and is encoded by the *PRNP* gene, mediating essential processes in mammalian nervous system [12–14]. This protein is a scaffolding molecule and orchestrates signaling complexes on the plasma membrane, interacting with a plethora of ligands [15]. Beyond its important physiological functions [16–18], PrP^C^ is also crucial in different cancer types [19, 20], including brain tumors. PrP^C^ is often found upregulated in GBM patient samples, and its silencing leads to diminished tumor growth and better survival expectancy in mice [21]. Moreover, PrP^C^ modulates the expression of stemness and differentiation markers in GSCs, as well as cellular migration, proliferation, self-renewal, and tumorigenicity [22, 23]. Despite these recent advances, a deeper comprehension of the molecules and signaling pathways modulated by PrP^C^ in GBM biology is still lacking and might elucidate novel therapeutic approaches against this glioma.

Herein, we analyzed four publicly available RNA sequencing (RNA-seq) datasets generated from patient-derived GBM at both bulk and single-cell (scRNA-seq) levels to unravel PrP^C^ functions in the biology of this tumor. A fifth public dataset consisting of proteomics data also from patient-derived GBM confirmed and strengthened our findings at the protein level. We also developed *GBMdiscovery*, an R-based user-friendly software application that will help researchers understand the impact of their genes of interest in GBM biology through the analysis of all RNA-seq datasets investigated in this work.

## Results

### Pathways associated with membrane-enclosed organelles and secretion are enriched in GBM cells with high* PRNP* expression

**Fig. 1:**
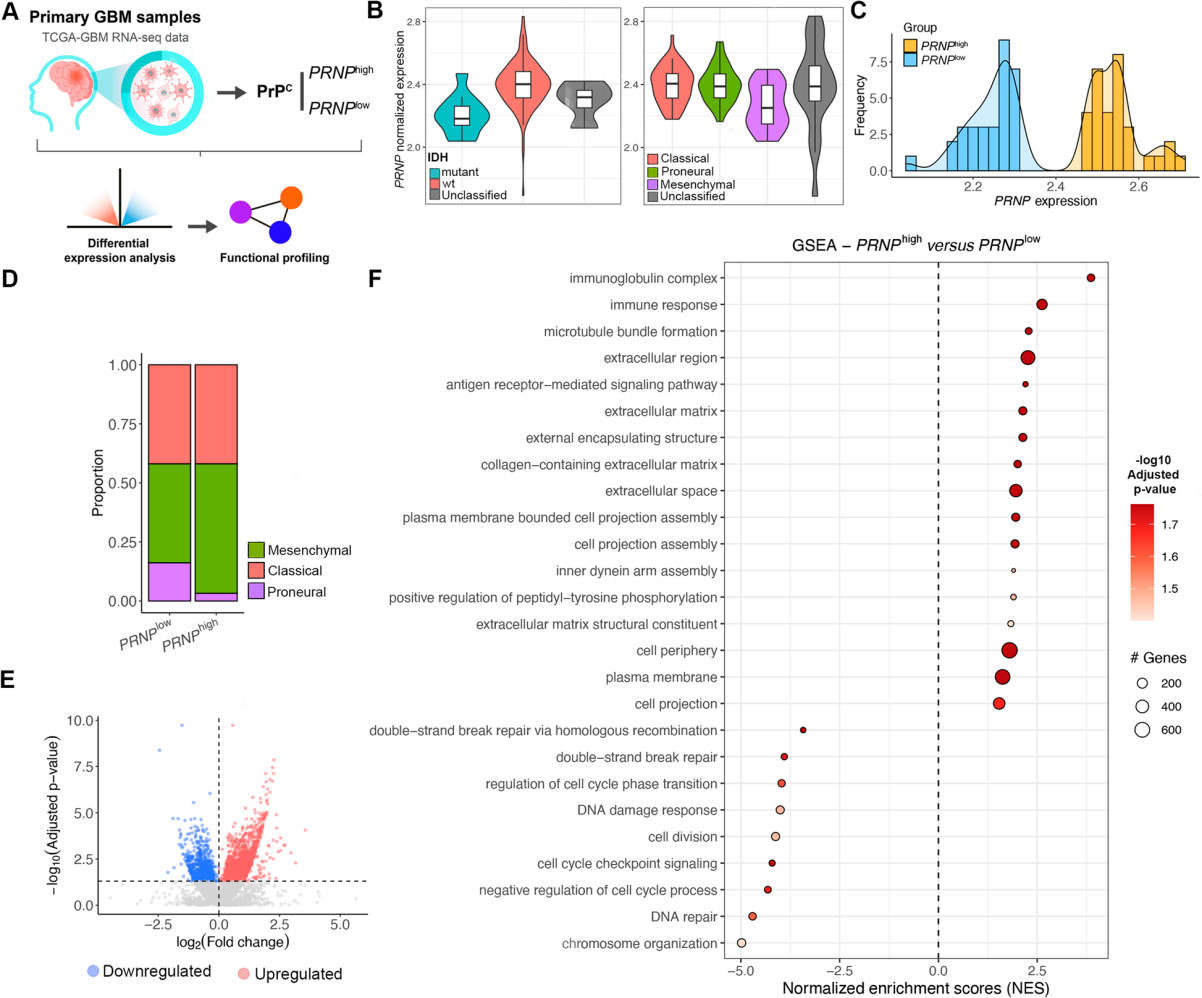
Impact of *PRNP* expression at bulk resolution in GBM samples from TCGA. **A** Schematic workflow of bulk RNA-seq data analyses of patient-derived primary GBM samples from TCGA (*n*=157). **B** Violin plots of *PRNP* normalized expression, according to log10 (counts per million [CPM]+1), in samples classified as IDH-mutant (*n*=11), IDHwt (*n*=142), or unclassified (*n*=4) (left, *p*=0.00016); and samples classified as classical (*n*=50), mesenchymal (*n*=67), proneural (*n*=18), or unclassified (*n*=22) (right, *p*=0.01). Kruskal-Wallis test. **C** GBM samples (*n*=124, after filtering) were ranked according to quartiles of *PRNP* expression. Those below the lower (*PRNP*^low^, *n*=31) and above the upper quartile (*PRNP*^high^, *n*=31) were selected, as shown in the density plot of *PRNP* expression. **D** GBM molecular subtype composition of the *PRNP*^low^ and *PRNP*^high^ groups. **E** Volcano plot of upregulated (red) and downregulated (blue) transcripts in *PRNP*^high^ relative to *PRNP*^low^. (The *PRNP* was removed from the plot). **F** Gene set enrichment analysis (GSEA) show enriched terms (Gene Ontology, GO) in the upregulated and downregulated transcripts of *PRNP*^high^ GBM, sorted by normalized enrichment score (NES) and colored by adjusted *p*-value

To elucidate the repertoire of biological phenomena that might be modulated by *PRNP*/PrP^C^ in GBM, we analyzed bulk RNA sequencing (RNA-seq) data of patient-derived primary GBM samples (n=157) from The Cancer Genome Atlas (TCGA) database (Fig. 1A). We initially verified that *PRNP* expression levels were increased in IDHwt GBM relative to IDH-mutant (Fig. 1B, left). Regarding molecular subtypes, *PRNP* presented similar expression levels between classical and mesenchymal GBM, and lower expression in the proneural subtype (Fig. 1B, right). In accordance with recent GBM classification guidelines, IDH-mutant (*n*=11) and samples of unknown subtype (*n*=22) or IDH status (*n*=4) were excluded prior to running any downstream analyses. Some unclassified samples in GBM subtype overlapped with unclassified IDH statuses, leading to 124 samples after filtering. We then normalized the count data of the remaining primary tumor samples according to log10(CPM+1) and ranked them by *PRNP* expression. Selecting GBM samples below the lower quartile to be the *PRNP*^low^ group (*n*=31), while samples above the upper quartile composed the *PRNP*^high^ group (*n*=31) (Fig. 1C). Next, we assessed the composition of each group regarding GBM molecular subtypes (Fig. 1D). We found that both groups presented a similar composition of classical samples, while *PRNP*^high^ consisted mainly of the mesenchymal subtype, with a decreased proportion of proneural samples relative to *PRNP*^low^ (Fig. 1D).

We used raw counts as input in DESeq2 [24] to identify differentially expressed transcripts (DETs) between the groups, using *PRNP*^low^ as control. In total, 2634 significant DETs were identified, of which 1749 were upregulated and 885 downregulated (Fig. 1E, Table S1). We subjected DETs to gene set enrichment analysis (GSEA) and observed that several terms (Gene Ontology, GO) associated with cell cycle and DNA damage response (including negative regulation of cell cycle process, cell cycle checkpoint signaling, cell division, double-strand break repair, and DNA repair) were downregulated in *PRNP*^high^ (Fig. 1F, Table S2). On the other hand, terms related to different aspects of membrane-enclosed organelles (including inner dynein arm assembly, microtube bundle formation, external encapsulating structure), plasma membrane events (cell projection, cell periphery, cell projection assembly, plasma membrane-bound cell projection assembly, plasma membrane) and secretion (extracellular region, extracellular matrix, collagen-containing extracellular matrix, extracellular space, extracellular matrix structural constituent) were upregulated in *PRNP*^high^ (Fig. 1F, Table S2). Additionally, over-representation analysis (ORA) of upregulated transcripts showed enrichment for extracellular transport and the trans-Golgi network (Table S3). This caught our attention because, in addition to being a critical scaffolding protein on the plasma membrane [25], PrP^C^ has been shown to be involved in intracellular trafficking and exosome biogenesis [26]. Moreover, many processes associated with the immune response (immunoglobulin complex, immune response, antigen receptor-mediated signaling pathway, among other terms) were substantially enriched in the *PRNP*^high^ group, in line with results that show an increase in the mesenchymal molecular subtype in these samples [27]. Overall, the initial bulk RNA-seq data assessment suggests that high *PRNP* expression might be associated with intra- and extracellular transport pathways.

### *PRNP*-positive cells are associated with vesicle dynamics in patient-derived GBM at single-cell level

**Fig. 2:**
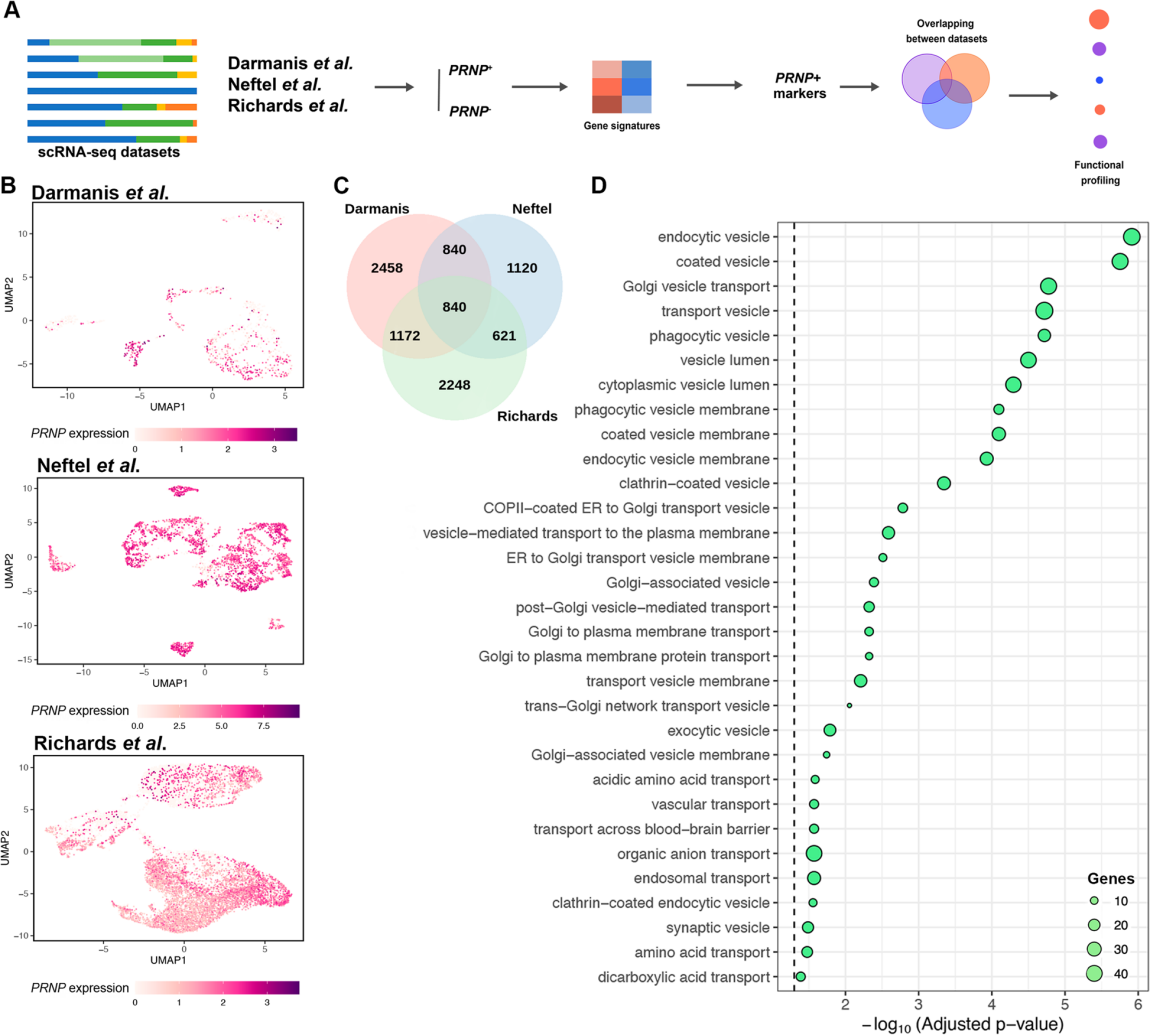
Integrated analysis of single-cell GBM datasets shows the impact of *PRNP* expression. **A** Schematic workflow of single-cell RNA-seq (scRNA-seq) data analyses carried out on three independent and publicly available datasets. **B** UMAP representation of *PRNP* expression in GBM cells from Darmanis et al., Neftel et al., and Richards et al. **(C)** Venn diagram of common marker genes of *PRNP*^+^ cells in all scRNA-seq datasets. **D** Functional profiling (ORA, GO) of the 840 common genes found in (C), ranked by -log_10_ (Adjusted *p*-value)

While bulk RNA-seq can provide remarkable insights to biological questions, it has the limitation that information on individual cells is lost, and only the average transcriptional profile of the whole sample is obtained [28]. Our observations from bulk transcriptomics prompted us to ascertain the extent of our findings at the single-cell level, exploring three publicly available patient-derived GBM single-cell RNA-seq (scRNA-seq) datasets from independent studies [4, 29, 30] (Fig. 2A). For each dataset, we first isolated the malignant from the non-neoplastic cells based on each study’s metadata, filtering out IDH-mutant, recurrent, pediatric, and unclassified samples. Then, we visualized how *PRNP* expression was distributed across the tumors (Figs. 2B and S1A and B), finding that *PRNP* is widely expressed in cancer cells, and its levels are heterogeneous among different samples.

Using a similar approach as in our bulk RNA-seq analysis, we classified the single-cells as *PRNP*=0 (*PRNP*^-^) or *PRNP*>0 (*PRNP*^+^). Marker genes of *PRNP*^+^ cells were identified and subjected to ORA for each dataset, resulting in several over-represented vesicle-related terms (Fig. S2). Next, we compared common *PRNP*^+^ marker genes among the studies. Our results show that 840 genes lay at the intersection of the three datasets (Fig. 2C, Table S4), and, once again, numerous vesicle-associated terms were enhanced among the intersecting genes (Fig. 2D, Table S5). In addition, endoplasmic reticulum (ER)- and Golgi-associated terms, structures intrinsically involved in intracellular traffic, were also enriched (Fig. 2D, Table S5). Altogether, our findings from single-cell transcriptomics provide an in-depth insight into the association of *PRNP* and vesicle biology in GBM.

### *PRNP* positively correlates with vesicle-associated genes in GBM

**Fig. 3:**
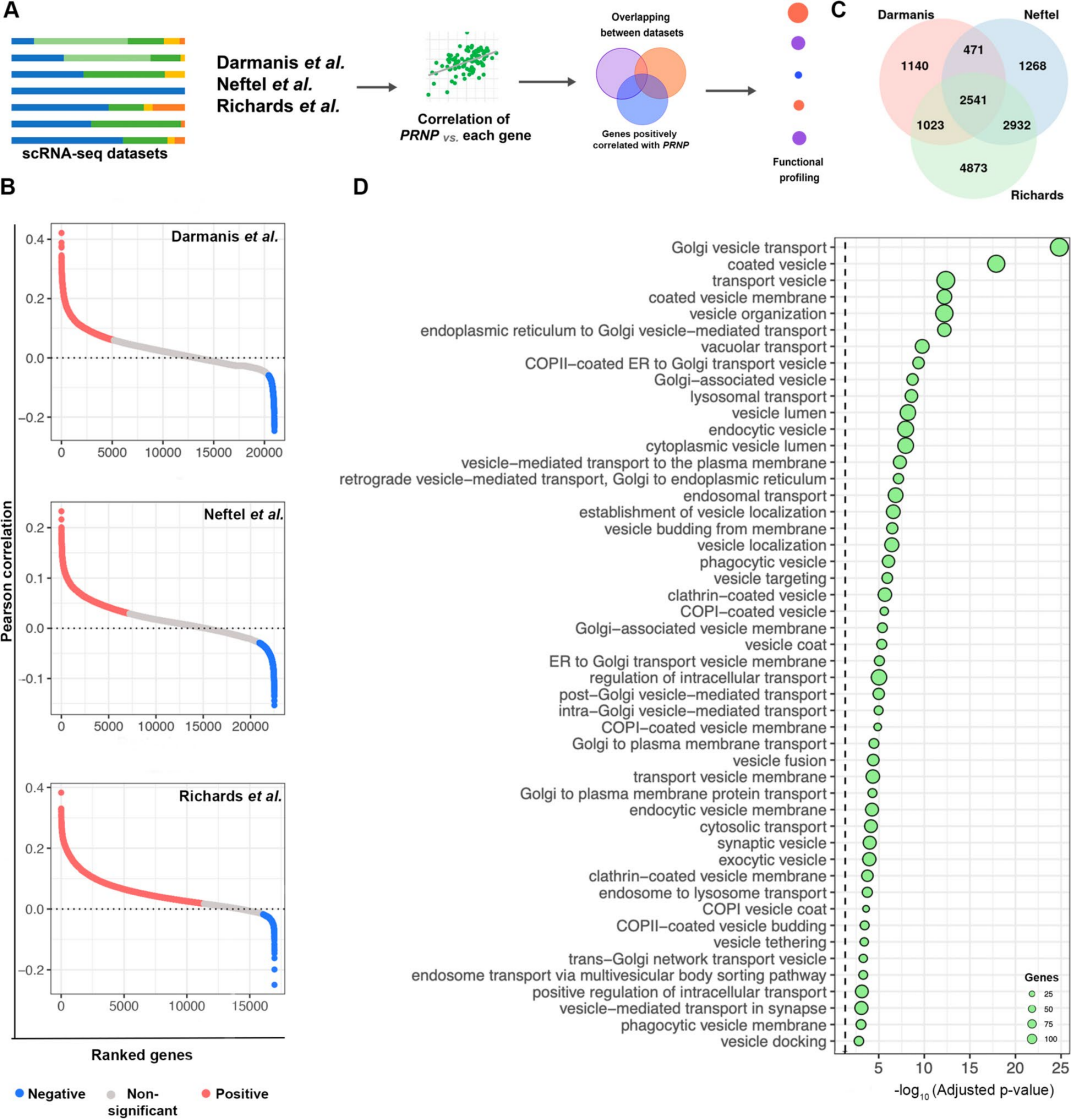
*PRNP* positively correlates with vesicle-associated genes at single-cell resolution. **A** Schematic workflow of the correlation analysis between *PRNP* expression and all genes identified in the scRNA-seq of the three independent patient-derived GBM datasets. **B** Pearson correlation shows significant positively (red) and negatively (blue) correlated genes with *PRNP* for the three scRNA-seq datasets. **C** Venn diagram of common positively correlated genes identified in (B). **D** ORA analysis (gProfiler2 and GO) of genes positively correlated with *PRNP*

Subsequently, we aimed to understand how *PRNP* expression correlates with the levels of other genes expressed by cancer cells (Fig. 3A). We calculated Pearson correlation between *PRNP,* and all the genes identified in the sequencing of each study (Fig. 3B), and compared the positively correlated genes between the datasets, finding 2541 common genes (Fig. 3C, Table S6). Besides vesicle biogenesis, processing, intra- and intercellular trafficking, secretion, budding, fission and fusion, a plethora of components of the endocytic pathway are also enriched among the common positively correlated genes (Fig. 3D, Table S7). These data reinforce the putative function of *PRNP*/PrP^C^ in the interplay between membrane-bound compartments and cellular trafficking.

**Fig. 4:**
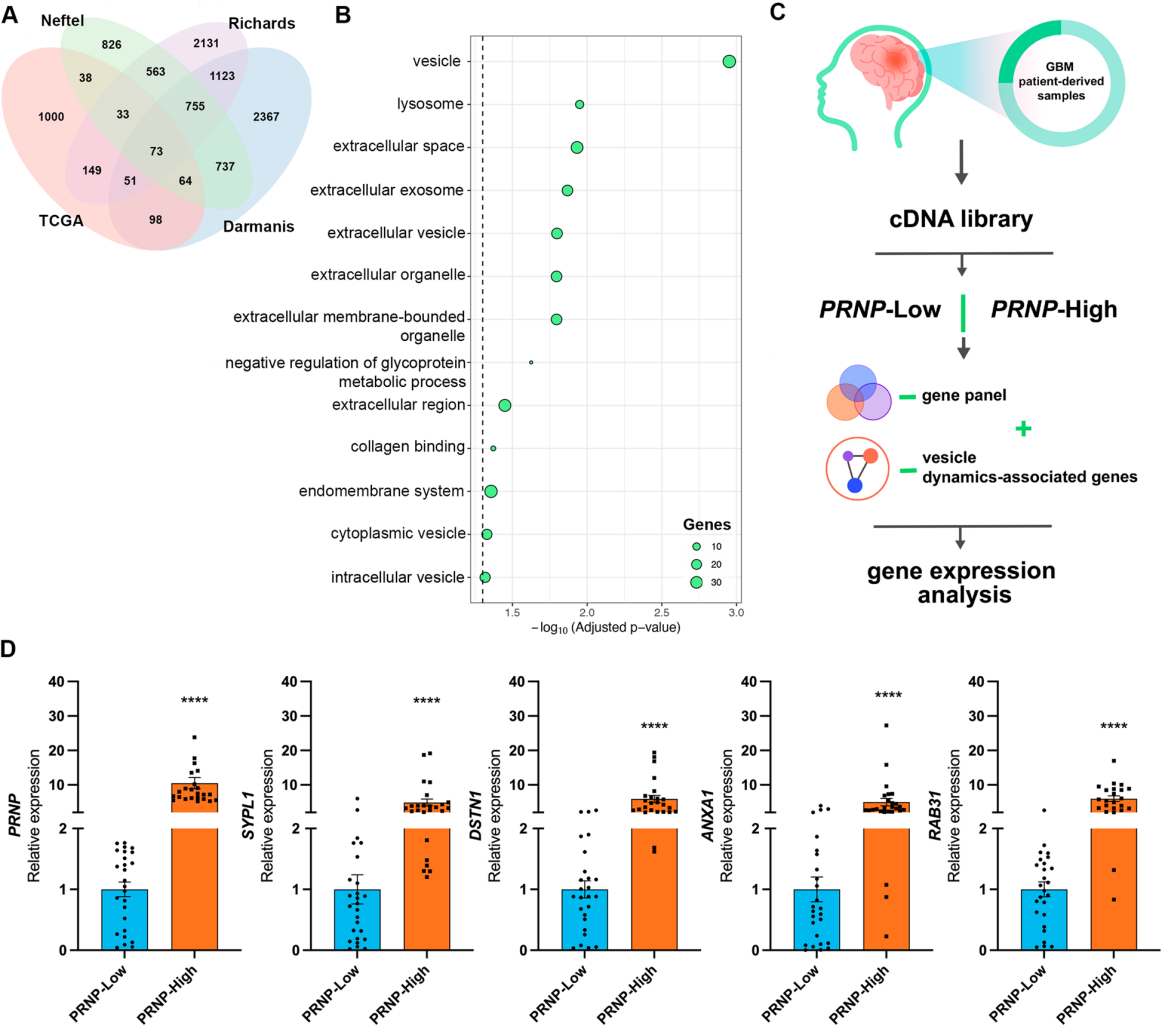
Identification of a 73-gene panel enriched in traffic-related structures and vesicle dynamics in *PRNP*^high^ samples and *PRNP*^+^ GBM cells. **A** Venn diagram of the 73 common genes with increased expression levels in *PRNP*^high^ GBM cells (TCGA bulk RNA-seq) and upregulated in *PRNP*^+^ cells (Darmanis et al., Neftel et al. and Richards et al.). **B** Over-representation analysis (gProfiler2 - GO, KEGG, Reactome, and WikiPathways) of common genes found in (A). **C** Schematic workflow of the analysis of an in-house cohort of GBM patient-derived samples. **D** Relative expression of *PRNP*, *SYPL1*, *DSTN*, *ANXA1* and *RAB31* in patient-derived GBM samples expressing Low (*n*=26) or High (n=26) *PRNP* levels by RT-qPCR and 2^-ΔΔCt^, normalized by *HPRT, GUSB and TBP*. Student *t*-test, ****p<0.0001

To strengthen our conclusions, we combined the findings from the analyzed bulk- and single-cell transcriptome data. We identified which genes were markers of *PRNP*^+^ GBM cells at the intersection among all scRNA-seq datasets and were also upregulated in the *PRNP*^high^ samples from TCGA bulk RNA-seq data. Our approach yielded a panel of 73 genes, one of which was *PRNP* (Fig. 4A, Table S8). In general, the genes from our panel displayed a higher expression in tumor samples (primary and recurrent) compared to non-neoplastic tissues (Fig. S3), and increased levels in IDHwt GBM compared to IDH-mutant (Figure S4). Among molecular subtypes, the genes from our 73-gene panel had a more heterogeneous expression level but presented a prominent enhancement in the mesenchymal subtype (Figure S5). Confirming the observations from previous analyses, functional profiling of this gene panel demonstrated that vesicle biology terms (vesicle, extracellular vesicle, extracellular exosomes, intracellular vesicle, among others), were enriched (Fig. 4B, Table S9). These findings further corroborate the association between *PRNP* expression and vesicle dynamics in GBM biology, highlighting a set of genes whose expression could be affected by *PRNP* levels in the tumor.

As a proof-of-concept, we used an in-house collection of patient-derived GBM samples to validate the expression levels of a set of genes from our 73-gene panel. For this selection, we searched the literature for previous experimental evidence indicating that the genes from our panel were related to intracellular traffic, endocytic or exocytic vesicles, and cytoskeleton maintenance, which is also relevant for organelle transport. Besides *PRNP*, we selected synaptophysin Like 1 (*SYPL1*) [31] and destrin (*DSTN*) [32] for gene expression quantification. Additionally, we also selected annexin A1 (*ANXA1*) [33] and Ras-related protein Rab-31 (*RAB31*), genes that are not shown in our panel but are noteworthy vesicle components and regulators of transport vesicles, including in GBM [34, 35]. We performed RT-qPCR on 104 patient-derived GBM, dividing them according to their *PRNP* expression. Briefly, we used the Δ-Cq values for normalized *PRNP* expression and stratified the samples into quartiles according to these values, using the samples above the upper quartile and below the lower quartile to define, respectively, high (*n*=26) and low (*n*=26) *PRNP* levels (Fig. 4C). Expression of *SYPL1, DSTN, ANXA1* and *RAB31* was increased in samples with high *PRNP* expression compared to samples with low *PRNP* expression (Fig. 4D). Therefore, our experimental results using an additional cohort of patient-derived samples corroborate our findings from RNA-seq analyses.

### Patient-derived whole-proteome analysis shows that PrP^C^ expression is associated with vesicle dynamics

To investigate whether our results would be corroborated beyond the transcriptional level, we analyzed a publicly available patient-derived GBM proteomics dataset [36]. We divided tumor samples into groups with either high (PrP^C^-high; *n*=13) or low PrP^C^ (PrP^C^-low; *n*=13) levels (Figure S6A) and identified differentially expressed proteins (DEPs) between these conditions, using PrP^C^-low as control (Figure S6B, Table S10). Protein classification shows membrane traffic proteins and cytoskeleton proteins among the upregulated DEPs (Figure S6C). Functional profiling revealed that several vesicle dynamics and traffic terms were enriched in the upregulated proteins in PrP^C^-high tumors (Figure S6D), which was in accordance with our previous transcriptomics data. Together, our findings advocate for an association of PrP^C^ with vesicle phenomena not only at transcriptional but also at protein levels.

### Vesicle dynamics signatures are positively correlated with *PRNP* expression in other patient-derived solid tumors

To evaluate if our findings extended to other cancers, we analyzed the impact of *PRNP* expression in other tumor types. We calculated the correlation between *PRNP* expression levels and vesicle dynamics signatures in different stages of patient-derived solid tumors from TCGA from: adrenal gland, bladder, breast, gallbladder, colon, head and neck, kidney, lung, ovary, pancreas, prostate, rectum, skin, stomach, esophagus, thyroid, and uterus (Figure S7, Table S11).

Our results showed that, for most cancer types, *PRNP* displayed significantly positive correlations with several vesicle dynamics signatures in at least one tumor stage. In particular, bladder, head and neck, kidney, and lung tumors were the cancer types with the highest number of positive correlations between *PRNP* expression and vesicle dynamics signatures among distinct tumor stages. These findings demonstrate, therefore, that *PRNP* expression is correlated with vesicle-related events in solid tumors from different sites, suggesting a conserved role of *PRNP* modulating these processes in cancer.

### Increased vesicle dynamics signatures associated with shortened overall survival of glioblastoma patients

**Fig. 5:**
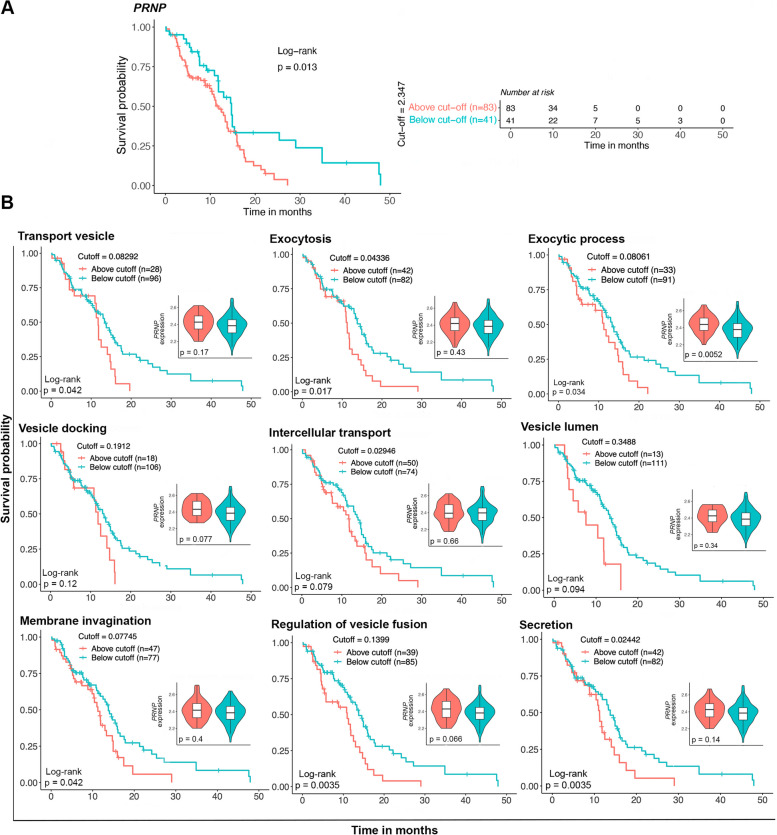
Impact of *PRNP* and vesicle dynamics signatures on patients’ overall survival. **A** Kaplan-Meier curves demonstrate the overall survival of GBM patients either below or above the optimal cutoff, calculated for *PRNP* expression levels. A risk table is shown at the left. Statistical significance was assessed using a log-rank test, and *p*-value is stated in the graph. **B** Kaplan-Meier curves demonstrate the overall survival of GBM patients either below or above the optimal cutoff, calculated for gene signatures of traffic- or vesicle-related processes (GO). Statistical significance was assessed using log-rank tests, and *p*-values are stated in the graphs. Inserts show *PRNP* normalized expression in samples above and under the cutoff of each gene signature

To understand the clinical relevance of our findings, our next step was to explore the association between *PRNP* expression and vesicle dynamics processes on patients’ overall survival, using TCGA-GBM survival data. We observed that a high *PRNP* expression is associated with a worse prognosis of GBM patients (Fig. 5A). Furthermore, we selected representative trafficking and vesicle dynamics gene sets from GO, based on our previous results. Subsequentially, we calculated gene signatures in IDHwt primary GBMs from TCGA, determining the optimal cutoff to separate samples with either low or high signatures, and performing Kaplan-Meier survival estimates (Fig. 5B).

The results demonstrate that enrichment for transport vesicle, exocytosis, exocytic process, vesicle docking, intercellular transport, vesicle lumen, membrane invagination, regulation of vesicle fusion and secretion is linked to shorter patients’ overall survival (Fig. 5B). Concomitantly with the survival curves, *PRNP* expression was evaluated in samples above and below cutoff for each signature and was significantly increased in exocytic process (Fig. 5B, inserts).

These results highlight the importance of our findings since PrP^C^ mRNA and protein levels are associated with biological phenomena that are likely to be responsible for poor survival expectancy for patients with GBM.

### *GBMdiscovery* as a novel platform to unravel the impact of specific genes in glioblastoma biology

**Fig. 6:**
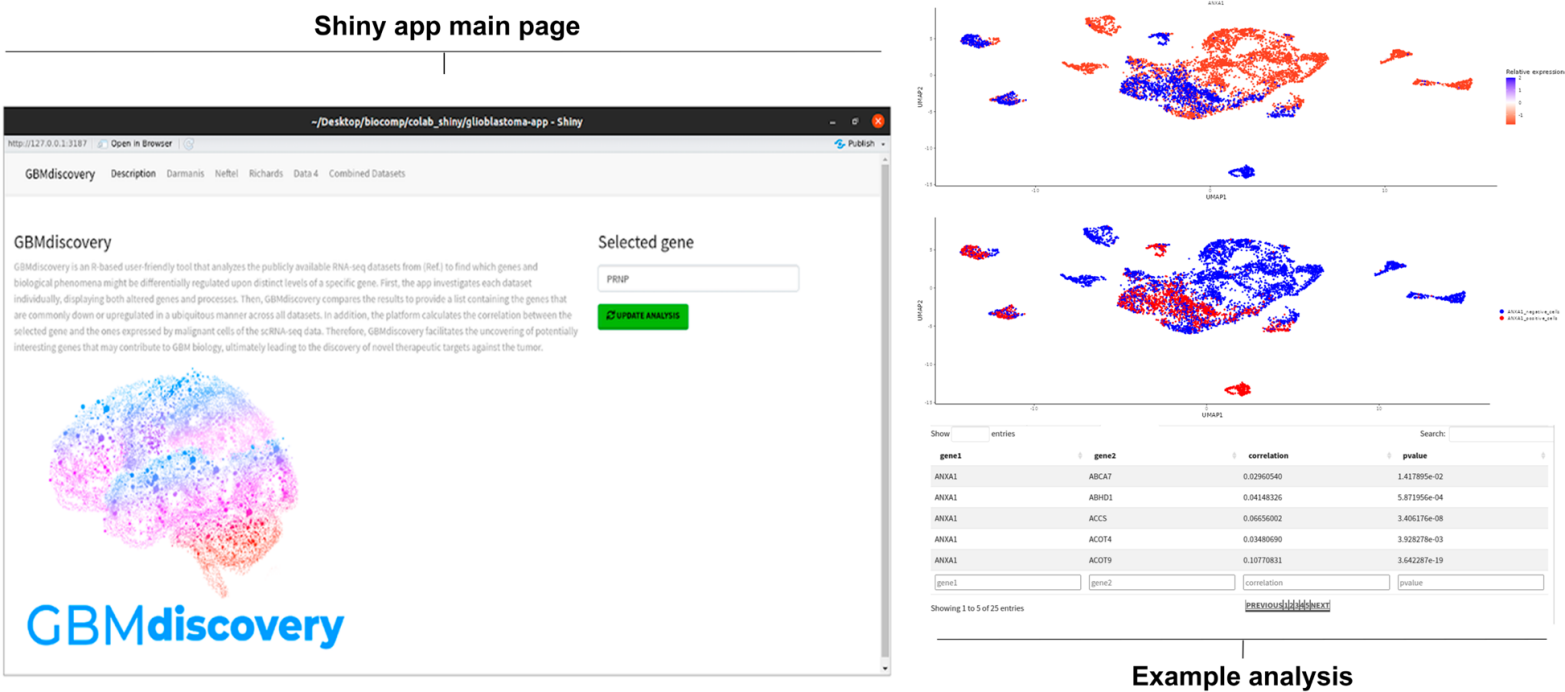
Leading page and example analysis of GBMdiscovery. GBMdiscovery is an R-based shiny app that allows users to discover the implications of their genes of interest in GBM biology, combining the four publicly available patient-derived bulk- and scRNA-seq datasets analyzed in the present study

We found a potential implication of PrP^C^ in GBM biology, demonstrating that its mRNA and protein levels are associated with intracellular traffic and, more specifically, vesicle dynamics in this tumor. Since transcriptomics provided such interesting biological insights, we developed GBMdiscovery, an R-based shiny app that allows users to discover the implications of their own genes of interest in GBM biology (Fig. 6). The app is user-friendly, allowing researchers from any area with no bioinformatics background to use it, and it can be downloaded from https://github.com/marilenehohmuth/GlioblastomaDiscovery.

*GBMdiscovery* analyzes all publicly available RNA-seq datasets presented in this study to find which genes and biological phenomena might be differentially regulated upon distinct levels of a specific gene. First, the app analyzes each dataset individually, displaying both genes and processes that are altered in each study, based on differential levels of the gene-of-interest of the user. GBMdiscovery then compares the results of all datasets to provide a list that contains the genes that are commonly down or upregulated upon distinct levels of the gene-of-interest in a ubiquitous manner across the four RNA-seq datasets. In addition, our app also calculates the correlation between this gene and all genes expressed by malignant cells identified in the studies. Therefore, GBMdiscovery aims to facilitate uncovering potentially interesting genes that may contribute to GBM biology, ultimately leading to the discovery of novel therapeutic targets against the tumor.

## Discussion

**Fig. 7:**
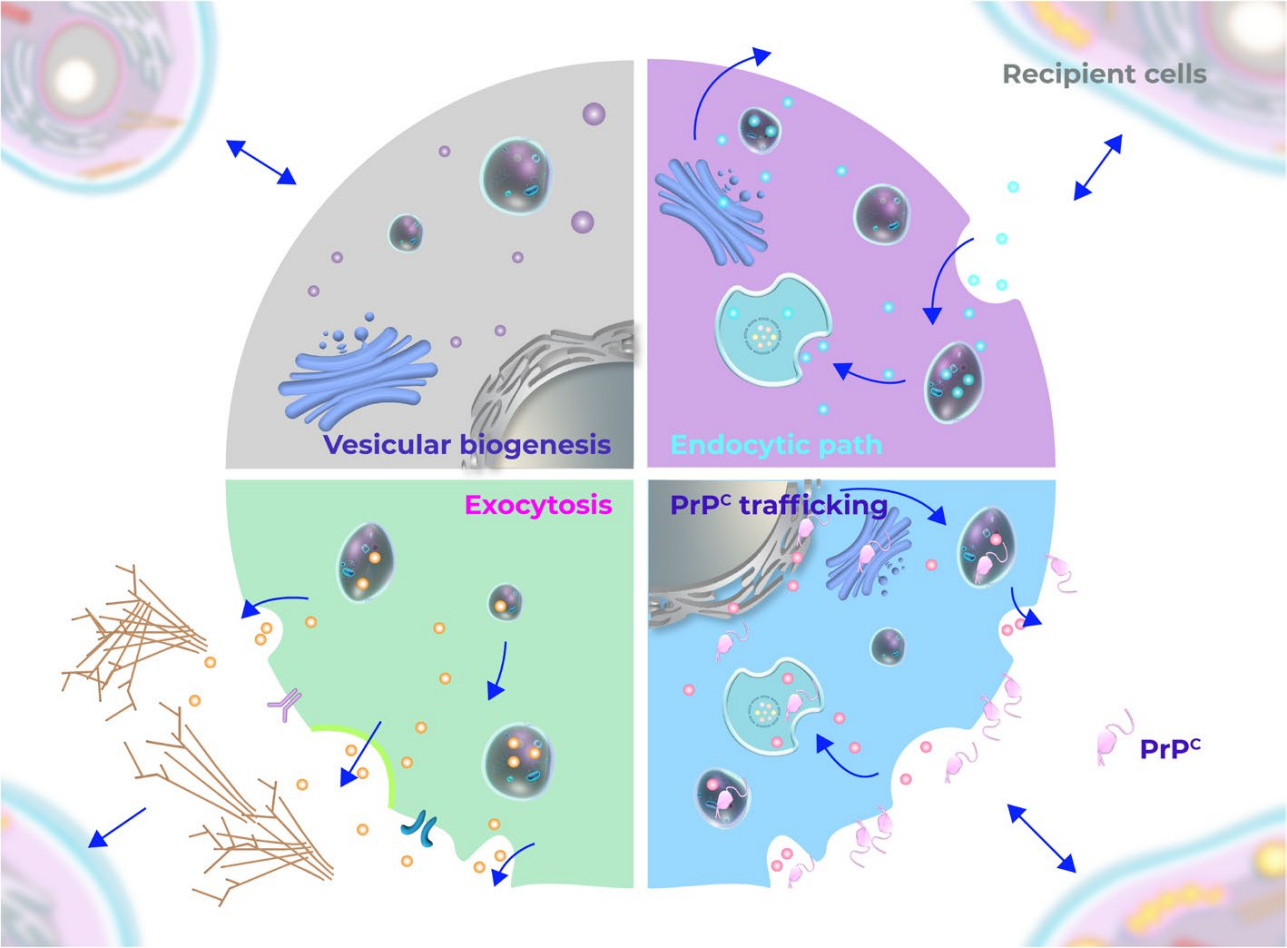
Schematic diagram of the mechanisms involved in extra- and intracellular trafficking by which PrP^C^ may affect GBM biology. PrP^C^ is transiently found in the ER and Golgi during its biogenesis and is internalized by a vesicle-mediated process to be recycled or degraded. Vesicular biogenesis, endocytic path and exocytosis are processes enriched in GBM cells with high *PRNP* expression

Intra- and intercellular trafficking are broad terms comprising diverse biological phenomena such as endocytosis, protein sorting, vesicle transport, and secretion, which play a crucial role in cell signaling and communication. While critical for physiological functions and development [37], these mechanisms are hijacked by neoplastic cells to promote tumor survival, progression, and spreading [38, 39]. These processes are known to contribute to GBM, the most aggressive form of brain tumor [40, 41]. Therefore, identification of molecules that orchestrate such pathways is imperative for developing new treatment strategies against this glioma. Herein, we described for the first time that intracellular traffic processes, particularly vesicle dynamics, are upregulated in GBM patient-derived samples/cells with high* PRNP*/PrP^C^ expression (Fig. 7). Moreover, we described a positive correlation between *PRNP* expression and the expression of genes associated with cellular trafficking and vesicle-related processes, proposing a putative role for this molecule in the control of GBM vesicle biogenesis, processing, and intra/inter-cellular transport.

Growing evidence has demonstrated that intracellular transport is essential for cancer metabolic reprogramming, modulating the energetic balance of cancer cells and their proliferative and invasive behaviors [39]. Regarding cell-cell interactions, exosomes have been implicated in exchanging a range of different cargoes between cancer cells and neoplastic and stromal cells [42]. The signal molecules carried from one cell to another ultimately lead to the regulation of pivotal mechanisms for tumor maintenance [38]. Exosomes have broad implications for cancer progression, as they contribute to forming pre-metastatic and metastatic niches [38, 42]. In GBM, EVs secreted by the tumor are enriched in pro-angiogenic factors. EVs derived from hypoxic GBM cells can promote tumor growth, vascularization, and proliferation, in addition to acidifying the tumor microenvironment, which leads to augmented EV trafficking both inwards and outwards the cells [43]. Small EVs released by GBM cells may be used for cancer subtyping since their cargo reflects the tumor phenotype and has been associated with enhanced glioma growth, proliferation, survival, migration, invasion, metabolic reprogramming, drug resistance, angiogenesis, and immunomodulation of the microenvironment [44]. In gliomas, microvesicles cargo can enhance tumor growth [45] transport molecules that modify gene expression in recipient cells [46], and trigger the proliferation of endothelial cells [47]. In the current study, we propose PrP^C^, a protein that regulates tumorigenesis and tumor growth [15, 21, 23], as an orchestrator of vesicle-related pathways in GBM.

In physiological conditions, previous studies have demonstrated that PrP^C^ regulates exosome secretion in astrocytes and fibroblasts [26] and participates in synaptic vesicle release [48]. PrP^C^ was implicated in the secretion of soluble factors by astrocytes and in their communication with neurons and extracellular matrix organization [49]. Evidence suggests this glycoprotein is sorted into exosomes and enriched in EVs [50]. Moreover, PrP^C^-expressing exosomes are tumorigenic and highly secreted by drug-resistant colorectal cancer cells upon hypoxic conditions in the tumor microenvironment [51]. The cellular location of PrP^C^ is dynamic. While it is mainly located on the cell surface, PrP^C^ is also found in other cellular compartments, such as ER and the Golgi apparatus, which are directly involved in intracellular traffic pathways [52, 53]. Additionally, this protein is observed in intracellular vesicles in different stages of endocytosis after being internalized [52]. Therefore, throughout its dynamic trafficking within the cell and leveraging its scaffolding features, PrP^C^ may interact with orchestrators of intracellular traffic and modulate vesicle dynamics mechanisms (Fig. 7). *PRNP*/PrP^C^ may also affect the expression of downstream genes and proteins involved in intracellular traffic phenomena, ultimately regulating vesicle-related processes, but this remains to be further investigated in vitro.

In this context, our work identified several genes whose expression is positively correlated with *PRNP* in GBM. From our 73-gene panel (*PRNP* + 72 genes), we assessed the expression of *PRNP*, *SYPL1* and *DSTN*, on a collection of patient-derived GBMs. *SYPL1* encodes synaptophysin-like 1 (SYPL1), a protein that has emerged as a prognostic marker and potential target in pancreatic ductal adenocarcinoma [31], hepatocellular carcinoma [54], and colorectal cancer [55]. Additionally, SYPL1 was described in the transport of vesicles [31]. *DSTN*, in turn, encodes destrin, a regulator of actin depolymerization [32]. The function of destrin in actin cytoskeleton maintenance is relevant from the vesicular biology perspective since the cytoskeleton plays key roles in vesicle transport [56, 57]. We also assessed the expression of *ANXA1* and *RAB31,* since these are relevant molecules already involved in vesicle phenomena in GBM. Specifically, *ANXA1* encodes the membrane protein annexin A1 (ANXA1), which is involved in tethering and EV aggregation [33]. *ANXA1* is a marker gene of mesenchymal states in GBM [4], which is consistent with, as we showed, *PRNP*^high^/*PRNP*^+^ cells being mainly classified as mesenchymal. *ANXA1* is overexpressed in high grade GBM samples and is a pivotal molecule to regulate GBM cell proliferation, migration, and invasion [34]. Moreover, increased expression level of *ANXA1* in gliomas was associated with worse prognosis [58]. The protein encoded by *RAB31* acts both as a driver of intraluminal vesicle formation and as a suppressor of multivesicular body degradation, ultimately regulating the exosome pathway independent of the endosomal sorting complex required for transport [59]. Interestingly, *RAB31* knockdown significantly affected the capacity of GBM cell line growth in animal models [60]. Furthermore, a recent study reports that *RAB31* silencing attenuates glioma invasion, process mediated by EVs from glioma-derived endothelial cells [35].

Interestingly, we stablished that our findings present clinical relevance, since we showed worse prognosis in patient-derived samples with both enhanced vesicle biology signatures and high *PRNP*/PrP^C^ expression. Moreover, as reported here, the association between *PRNP* and vesicular dynamics has a potential impact in the biology of several types of solid tumors, strengthening this pathway as putative novel target for cancer therapeutic strategies.

Altogether, our findings provide valuable resources to further clarify molecular mechanisms governed by PrP^C^ in tumor biology and open new paths for future GBM research. Of great interest, we make available a novel user-friendly tool that will expand our knowledge on this glioma, allowing users to evaluate the impact of their genes of interest in GBM biology in a robust manner across various RNA-seq datasets at both bulk and single-cell resolutions.

## Methods

### Bulk RNA sequencing data analysis

#### Data retrieval

GBM bulk RNA-seq data and corresponding metadata were retrieved from TCGA through TCGAbiolinks (v2.30.0) in R (v4.3.2). We downloaded raw count data of 175 samples of the TCGA-GBM project, of which 157 corresponded to primary tumor samples, 13 to recurrent samples, and 5 to solid normal tissue. To assess *PRNP* expression across different IDH statuses and transcriptional subtypes, the raw counts of all primary tumors were normalized according to counts per million (CPM) using edgeR (v3.42.4) [61] and converted to the log_10_ (CPM+1) scale. After that, IDH-mutant samples, and samples whose IDH status or transcriptional subtype was unknown were removed from the cohort; 124 samples remained in the dataset.

#### Establishment of groups with distinct *PRNP* expression levels

Following the same procedure described above, we obtained log_10_ (CPM+1)-normalized counts for all 124 primary IDHwt GBMs. Thereafter, we found the quartiles of *PRNP* expression across the samples, classifying those below the lower quartile – which displayed decreased *PRNP* levels – as *PRNP*^low^ (*n*=31) and the ones above the upper quartile – with increased *PRNP* expression – as *PRNP*^high^ (*n*=31).

#### Identification of differentially expressed transcripts between *PRNP*^high^ and *PRNP*^low^ samples

After obtaining groups with different *PRNP* expression levels, we used the raw count data of the *PRNP*^high^ and *PRNP*^low^ groups as input in DESeq2 (v1.40.2) [24] for differential expression analysis. *PRNP*^low^ was used as the control group. Log fold change shrinkage was performed with the shrinkage estimators of a normal distribution. Differentially expressed transcripts (DETs) satisfying p_adjusted_<=0.05 were considered significant and selected for downstream analysis.

#### Functional profiling

We subjected the significant DETs to functional profiling with clusterProfiler (v4.10.0). Gene set enrichment analysis (GSEA) was performed with clusterProfiler’s gseGO function using the Gene Ontology (GO) database. All GO’s categories ("BP”, biological processes; “CC”, cellular components; and “MF”, molecular functions) were considered. The correction of *p*-values was done with the Benjamini-Hochberg (BH) method and GO terms whose p_adjusted_<=0.05 were considered significant. Over-representation analysis (ORA) was carried out with clusterProfiler’s enrichGO function with the same settings and significance threshold specified for GSEA.

### Single-cell RNA sequencing data analysis

#### Data retrieval

GBM single-cell RNA-seq (scRNA-seq) data were retrieved from three distinct sources. First, we downloaded raw count data and corresponding metadata of 3589 single cells from the work of Darmanis et al. (http://gbmseq.org); second, log-transformed, transcripts per million (TPM)-normalized data and associated information of 7930 single cells were obtained from the study of Neftel et al. via the Broad Institute Single-Cell Data Portal (https://singlecell.broadinstitute.org/single_cell/study/SCP393/single-cell-rna-seq-of-adult-and-pediatric-glioblastoma); and third, raw count data and related metadata of 44712 single cells were downloaded from the work of Richards et al. through Broad Institute’s Portal as well (https://singlecell.broadinstitute.org/single_cell/study/SCP503/gradient-of-developmental-and-injury-reponse-transcriptional-states-define-functional-vulnerabilities-underpinning-glioblastoma-heterogeneity).

#### Cell filtering

Cells from all three works had already been filtered by the authors of the respective studies regarding scRNA-seq data quality control metrics. Cell type classification of each cell was provided by the authors in the studies’ associated metadata. Here, we filtered cells according to their cell type assignments. As we aimed to investigate *PRNP* expression implications only in neoplastic cells, we isolated those malignant cells in the three datasets, resulting in 1091 cells for Darmanis et al. dataset; 6896 cells for Neftel et al. dataset; and 14207 cells for Richards et al. dataset. Recurrent tumors were excluded from the datasets, as well as pediatric samples and IDH-mutant tumors.

#### Dimensionality reduction

We processed the data of malignant cells from each of the three datasets using FUSCA (v1.3.1) [62]. For the Darmanis et al. and Richards et al. datasets, we normalized and scaled count data according to all genes, subsequently performing principal component analysis (PCA). In the case of the Neftel et al. dataset, as count data were already normalized, we skipped the normalization step and only scaled the data according to all genes, performing PCA afterwards. Uniform manifold approximation and projection (UMAP) was then employed for all three datasets, considering the 15 principal components with the highest standard deviations.

#### Correlation between *PRNP* and all genes expressed by glioblastoma cells

We calculated the Pearson correlation between *PRNP* expression and each gene’s expression levels for each dataset individually. Statistical significance was defined as *p*<=0.05. We compared the correlation results of each dataset and selected the genes that were positively correlated with *PRNP* in all three datasets (*n*=2541) for functional profiling. We performed ORA with clusterProfiler’s enrichGO. All GO’s categories were considered, and *p*-values were corrected with the BH method. Terms whose p_adjusted_<=0.05 were considered significant.

#### Classification of glioblastoma cells according to *PRNP *expression

Using normalized count data, we classified the malignant cells of each dataset according to their *PRNP* levels: those which displayed expression of this gene were assigned as *PRNP*^*+*^, while the ones that did not express *PRNP* were considered *PRNP*^-^.

#### Identification of marker gene signatures in *PRNP*^+^ and *PRNP*^-^ glioblastoma cells

Once we established groups of GBM cells either expressing *PRNP* or not in each dataset, we proceeded to the identification of marker gene signatures that characterized *PRNP*^+^ and *PRNP*^-^ cells. For each case, we utilized FUSCA’s findSignatures function to find marker genes using Wilcox statistical tests applying a cut-off of *p*<=0.05. Marker genes of *PRNP*^+^ and *PRNP*^-^ GBM cells of each dataset were subjected to GSEA and ORA with gseGO and enrichGO, respectively, using all categories of the GO database, with *p*-values being corrected with the BH method. Terms whose p_adjusted_<=0.05 were considered significant.

### Comparison of the results from each RNA sequencing dataset (bulk and single-cell)

We compared the upregulated transcripts in the *PRNP*^high^ group from TCGA bulk RNA-seq analysis with marker gene signatures of *PRNP*^+^ GBM cells from each scRNA-seq dataset. We subjected the 73 (*PRNP* + 72) genes that had higher expression in *PRNP*^high^/*PRNP*^+^ in all RNA-seq datasets to ORA with enrichGO with the same settings specified above. Since there were only a few genes in the gene panel, we also used gProfiler2 (v0.2.2) to perform ORA including GO and other databases, such as KEGG, Reactome, and WikiPathways.

### Survival analysis

#### Impact of* PRNP* expression on overall survival

We stratified the 124 primary IDHwt GBM samples into two groups based on *PRNP* expression levels using the optimal cut-off determined by maxstat.test (v0.7-25) in R. The optimal cut-off was calculated with log-rank statistics and the HL method for *p*-value approximation. Samples above the optimal cut-off were considered to have high levels of the gene of interest, whilst samples below that threshold were considered to have low levels. Finally, for Kaplan-Meier survival estimates, we used the survival (v3.5-7) and survminer (v0.4.9) packages.

#### Impact of gene set signatures on overall survival

We used log_10_(CPM+1)-normalized counts of all 124 primary IDHwt GBM samples to calculate gene set signature scores with gene set variation analysis (GSVA) [63]. GO gene sets were obtained from the Molecular Signatures Database (MSigDB) (v.7.5.1). We used the maxstat.test (v0.7-25) to calculate the optimal cut-off based on log-rank statistics and the HL method for *p*-value approximation. Samples above the optimal cut-off were considered to have high levels of the gene set of interest, whilst samples below that threshold were considered to have low levels. Finally, for Kaplan-Meier survival estimates, we used the survival (v3.5-7) and survminer packages (v0.4.9).

### Proteome analysis

#### Data retrieval, processing, and establishment of groups with different PrP^C^ levels

GBM proteomics data were obtained from the Proteomics Identifications Database (PRIDE). The selected dataset consisted of patient-derived GBMs (PRIDE Project PXD015545) [36], containing 12 sets with 6 samples each, tagged with 6 different tandem mass tags (TMT). The GBM Global sets 1 to 6 and 7 to 12 were selected for further analysis. Samples were filtered for contaminants, for reverse database, and the ones only identified by site. Next, samples were normalized by dividing each sample from each set by the Global Internal Standard (GIS) of the respective set. Following this, each ratio obtained by the normalization was log-transformed [log_2_(ratio)]. Then, the median of the samples was subtracted to centralize the normals in each sample, and with that, the groups of sets (1 to 6 and 7 to 12) were joined. Samples of normal tissue, which were 4, and the 12 GIS were removed, remaining 50 GBM samples (10 proneural, 10 neural, 14 classical, and 16 mesenchymal). Following this, samples were filtered according to PrP^C^ log_2_ (ratio) and separated into the PrP^C^-High (*n*=13) and PrP^C^-Low (*n*=13) expression groups, and subjected to differential expression analysis, using PrP^C^-Low as controls.

#### Differential expression analysis, overrepresentation analysis, and enrichment map visualization

For the comparison between the PrP^C^-High and PrP^C^-Low groups, the Benjamini-Hochberg method was used to determine differentially expressed proteins (DEPs) using PrP^C^-Low as control. In this analysis, we employed FDR≤0.05 and s0=0.1 as cut-offs. Upregulated DEPs in PrP^C^-High samples were subjected to ORA in g:Profiler using the g:SCS test to determine statistical significance and GO, Reactome, KEGG, and WikiPathways as gene set databases. A cut-off of *p*<0.05 was employed. ORA results were visualized through an enrichment map [64], which was built using the EnrichmentMap plugin in Cytoscape. Overrepresented terms were clustered with the AutoAnnotate plugin. Protein classes were analyzed using Panther database [65].

### Analysis of other solid tumors

Using TCGAbiolinks (v2.30.0), we retrieved the data of adrenocortical carcinoma (ACC), bladder urothelial carcinoma (BLCA), breast invasive carcinoma (BRCA), cervical squamous cell carcinoma and endocervical adenocarcinoma (CESC), cholangiocarcinoma (CHOL), colon adenocarcinoma (COAD), esophageal carcinoma (ESCA), head and neck squamous cell carcinoma (HNSC), kidney renal clear cell carcinoma (KIRC), liver hepatocellular carcinoma (LIHC), lung adenocarcinoma (LUAD), ovarian serous cystadenocarcinoma (OV), pancreatic adenocarcinoma (PAAD), pheochromocytoma and paraganglioma (PCPG), prostate adenocarcinoma (PRAD), rectum adenocarcinoma (READ), skin cutaneous melanoma (SKCM), stomach adenocarcinoma (STAD), and uterine corpus endometrial carcinoma (UCEC) samples from TCGA. In total, we retrieved the data of 19 solid tumor types other than GBM, spanning different organs and distinct locations of the human body. For each cancer type, we subset only primary tumors and normalized raw count data according to log_10_(CPM+1), calculating vesicle dynamics signature scores with GSVA based on selected GO gene sets from MSigDB (v7.5.1) afterwards. Then, for each tumor stage, we computed the Spearman correlation between *PRNP* expression and those vesicle dynamics signatures, considering *p*<=0.05 statistically significant.

### Experimental procedures

#### Samples

GBM samples were collected during the surgical procedure and immediately frozen in liquid nitrogen after resection by the group of Brain Tumors and Metastases of the Division of Neurosurgical Clinic of the Central Institute of the Department of Neurology of the Hospital das Clinicas of the School of Medicine of the University of Sao Paulo (HC-FMUSP). The casuistry of this project consisted of 103 GBMs confirmed by histopathological analysis of the HC-FMUSP Pathological Anatomy Division and are part of the biorepository samples collected during the Clinical Genome Project, approved by the National Ethics Commission (CONEP) and the Ethics Committee for Analysis of Research Projects (CAPPesq) of HC-FMUSP, under protocol number 830/01. Post-informed consents were obtained from all patients with tumor and epilepsy or from their legal guardians.

#### Total RNA extraction and cDNA synthesis

Frozen tumor samples were analyzed in 4μm cryosections and stained with hematoxylin and eosin for quality verification. Total RNA from tumors were also extracted with RNeasy Mini Kit. The quality and concentration of RNA were determined by measuring the absorbance in NanoDrop Spectrophotometers (Thermo Fisher Scientific) at 260 and 280 nm, and A260/A280 ratios greater than 1.8 were considered satisfactory for purity. cDNA synthesis was performed by reverse transcription with SuperScript III reverse transcriptase, RNase inhibitor (RNaseOUT), random oligonucleotides, and oligodT, according to the manufacturer's recommendations (Thermo Fisher Scientific). cDNA was treated with 1U RNase H (Thermo Fisher Scientific) at 37°C for 30 minutes and at 72°C for 10 minutes, diluted in Tris-EDTA buffer, and stored at -20°C for subsequent analysis of gene expression.

#### Quantitative real-time PCR

Gene expression levels in tissue samples were analyzed by reverse transcription quantitative real-time PCR (RT-qPCR). Reactions were performed by the incorporation method of SYBR Green on QuantStudio 3 (Thermo Fisher Scientific). The reactions were performed in duplicates in a final reaction volume of 10 μl, containing 5 μl of Power SYBR Green PCR Master Mix (Thermo Fisher Scientific), 2.5 μl of cDNA, and 2.5 μl of primers in a pre-standardized concentration. The primers used for the qPCR reaction were the following: *ANXA1* Forward: GCGGTGAGCCCCTATCCTA; Reverse: TGATGGTTGCTTCATCCACAC; *RAB31* Forward: GGGGTTGGGAAATCAAGCATC; Reverse: GCCAATGAATGAAACCGTTCCT; *DSTN* Forward: ATTTTGTGGGAATGCTTCCTGA; Reverse: GCATCCTTGGAGCTTGCATAG; *SYPL1* Forward: AAGATTACGTCCTCATAGGCGA; Reverse: TCGTGTAGCCAACATAAAGCAG; *PRNP* Forward: AGTCAGTGGAACAAGCCGAG; Reverse: CTGCCGAAATGTATGATGGGC. Amplification conditions were: initial incubation at 50°C for 5 minutes and at 95°C for 10 minutes, followed by 40 cycles of 95°C for 15 seconds and 60°C for 60 seconds. The expression value of the gene was normalized with reference genes as internal control: *HPRT* Forward: TGAGGATTT GGAAAGGGTGT; Reverse: GAGCACACAGAGGGCTACAA; *GUSB* Forward: GAAAATACGTGGTTGGAGAGCTCATT; Reverse: CCGAGTGAAGATCCCCTTTTTA; and *TBP* Forward: AGGATAAGAGAGCCACGAACCA; Reverse: CTTGCTGCCAGTCTGGACTGT. Single-product amplification was confirmed by analyzing its dissociation curve.

#### RT-qPCR analysis

One hundred three GBM samples were separated from non-tumoral tissue and classified based on Δ-Cq values for normalized *PRNP* expression, using the geometric mean of *TBP*, *HPRT,* and *GUSB* as endogenous controls. Specifically, samples were stratified into quartiles according to the normalized values, and samples lying above the upper quartile and below the lower quartile, respectively, high (*n*=26) and low (*n*=26) *PRNP* levels, were used for relative gene expression analysis, with *PRNP* low as the control group. Relative gene expression was assessed by the 2^-ΔΔCt^ method [66], followed by Student's *t*-test to assess the significance of the results.

### Supplementary Information

**Additional file 1: Figure S1.** 

**Additional file 2: Figure S2.**

**Additional file 3: Figure S3.**

**Additional file 4: Figure S4.**

**Additional file 5: Figure S5.**

**Additional file 6: Figure S6.**

**Additional file 7: Figure S7.**

**Additional file 8: Table S1.**

**Additional file 9: Table S2.**

**Additional file 10: Table S3.**

**Additional file 11: Table S4.**

**Additional file 12: Table S5.**

**Additional file 13: Table S6.**

**Additional file 14: Table S7.**

**Additional file 15: Table S8.**

**Additional file 16: Table S9.**

**Additional file 17: Table S10.**

**Additional file 18: Table S11.**

## Data Availability

All RNA-seq datasets analyzed in this study are publicly available for download. GBM single-cell RNA-seq (scRNA-seq) data were retrieved from: http://gbmseq.org; https://singlecell.broadinstitute.org/single_cell/study/SCP393/single-cell-rna-seq-of-adult-and-pediatric-glioblastoma; https://singlecell.broadinstitute.org/single_cell/study/SCP503/gradient-of-developmental-and-injury-reponse-transcriptional-states-define-functional-vulnerabilities-underpinning-glioblastoma-heterogeneity. Supplementary tables will be made available before publication. The scripts that were used for the analyses and the *GBMdiscovery* app will be made publicly available at https://github.com/marilenehohmuth/GlioblastomaDiscovery.
