# Supplementary figures and images for: Integrated transcriptomics uncovers an enhanced association between the prion protein gene expression and vesicle dynamics signatures in glioblastomas
Source: BMC Cancer. 2024 Feb 13;24:199. doi: 10.1186/s12885-024-11914-6 (PMC10863147; doi:10.1186/s12885-024-11914-6)

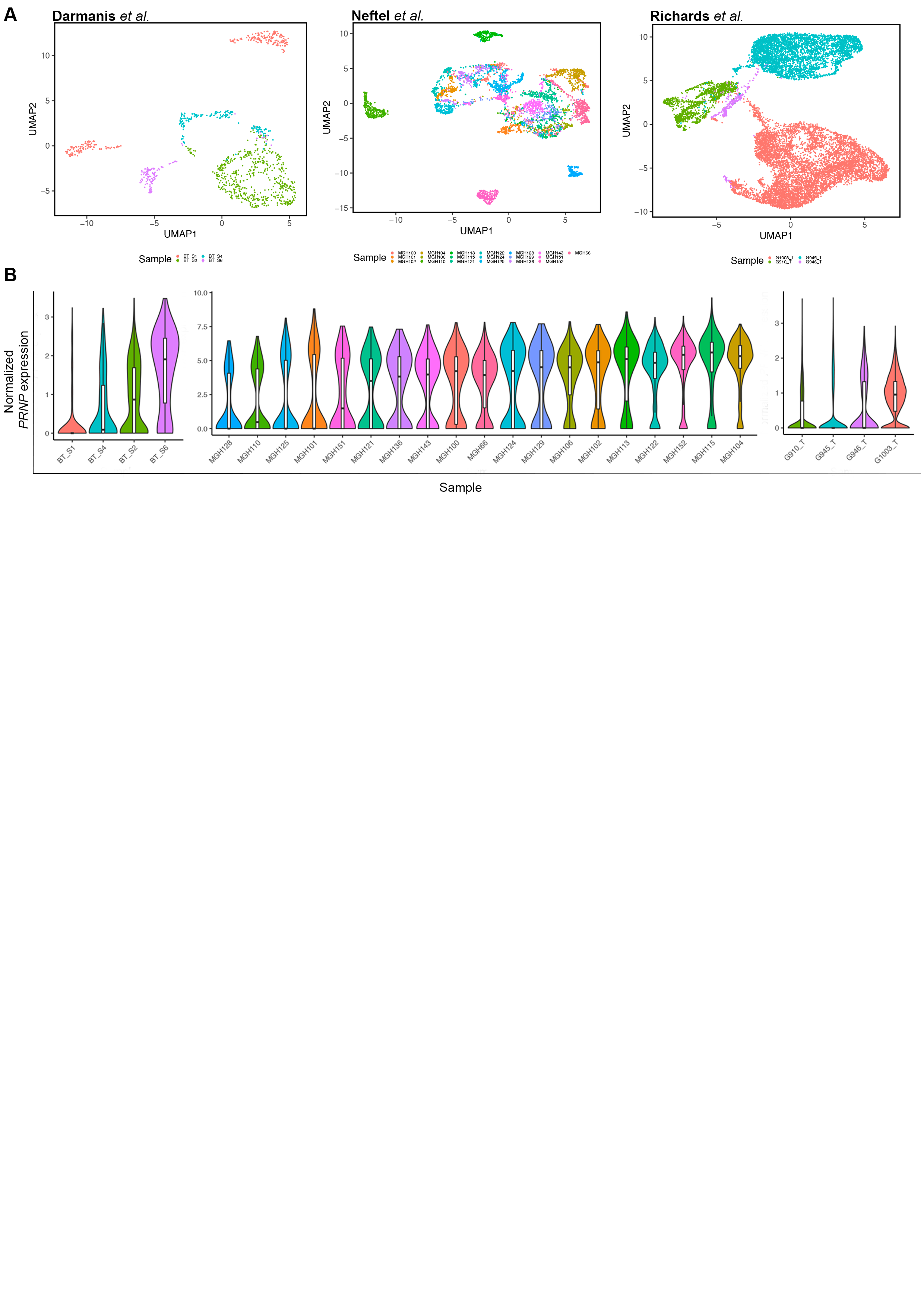

Supplement: Supplementary file 1 — Additional file 1: Figure S1. [file 12885_2024_11914_MOESM1_ESM.png]

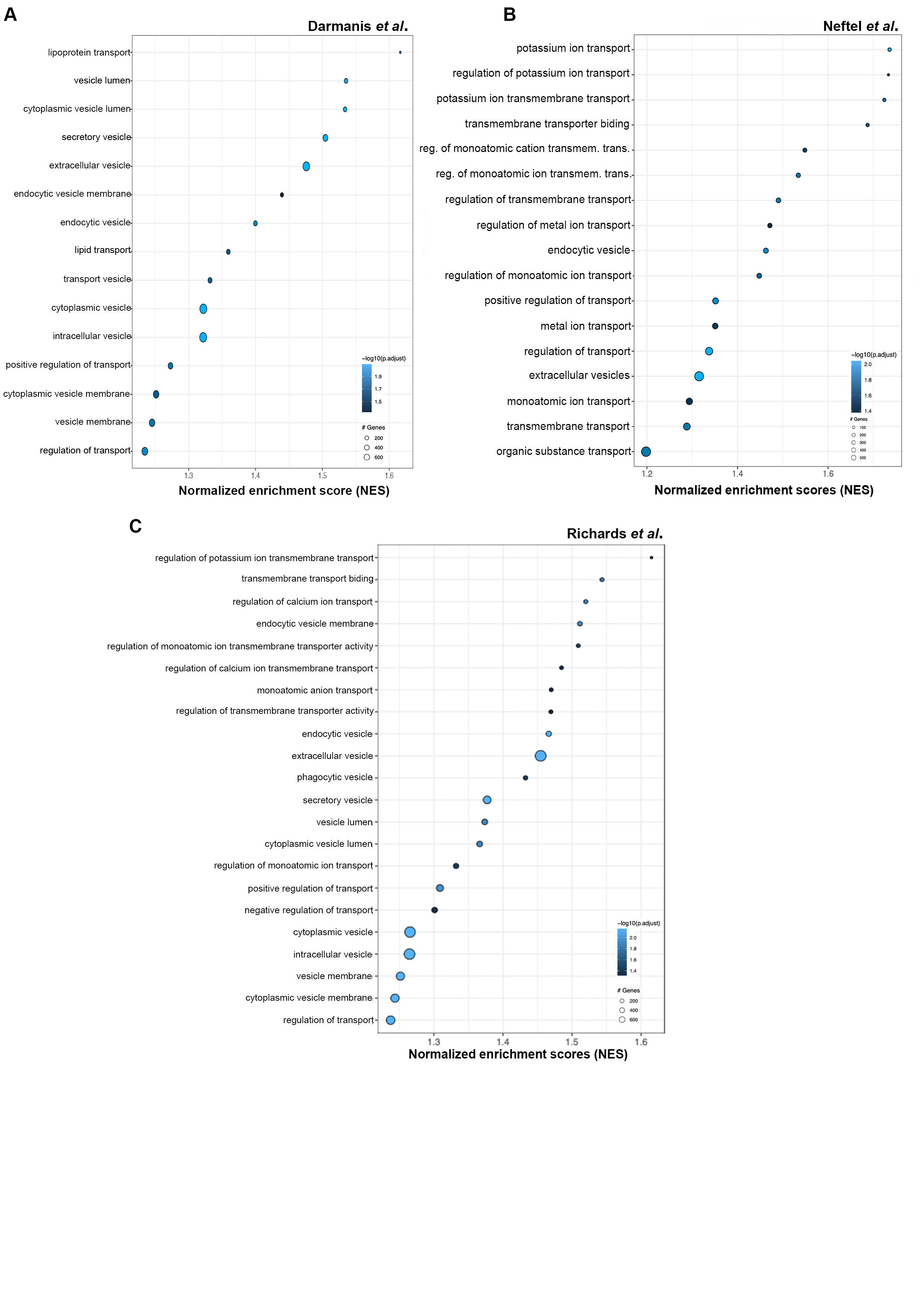

Supplement: Supplementary file 2 — Additional file 2: Figure S2. [file 12885_2024_11914_MOESM2_ESM.png]

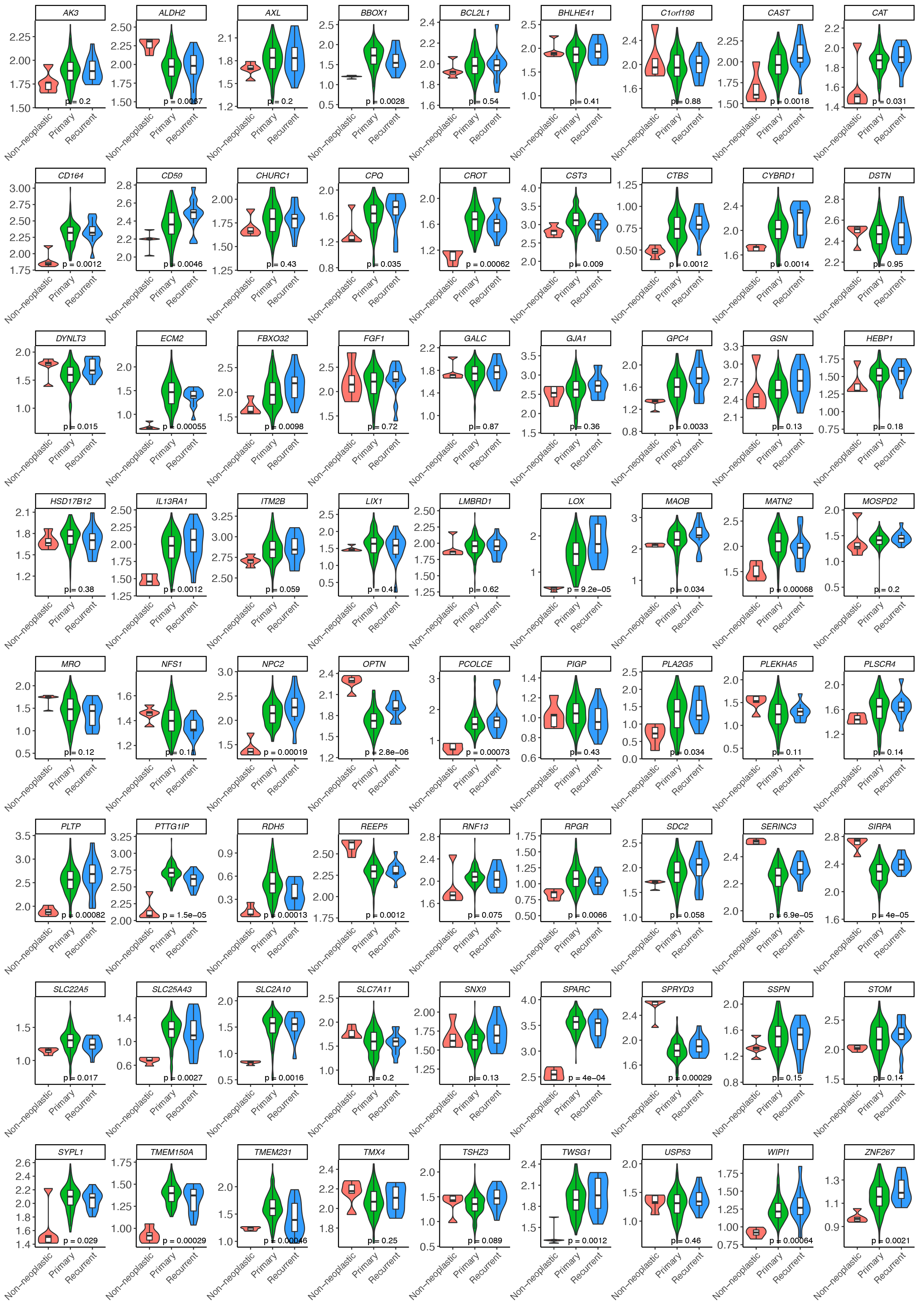

Supplement: Supplementary file 3 — Additional file 3: Figure S3. [file 12885_2024_11914_MOESM3_ESM.png]

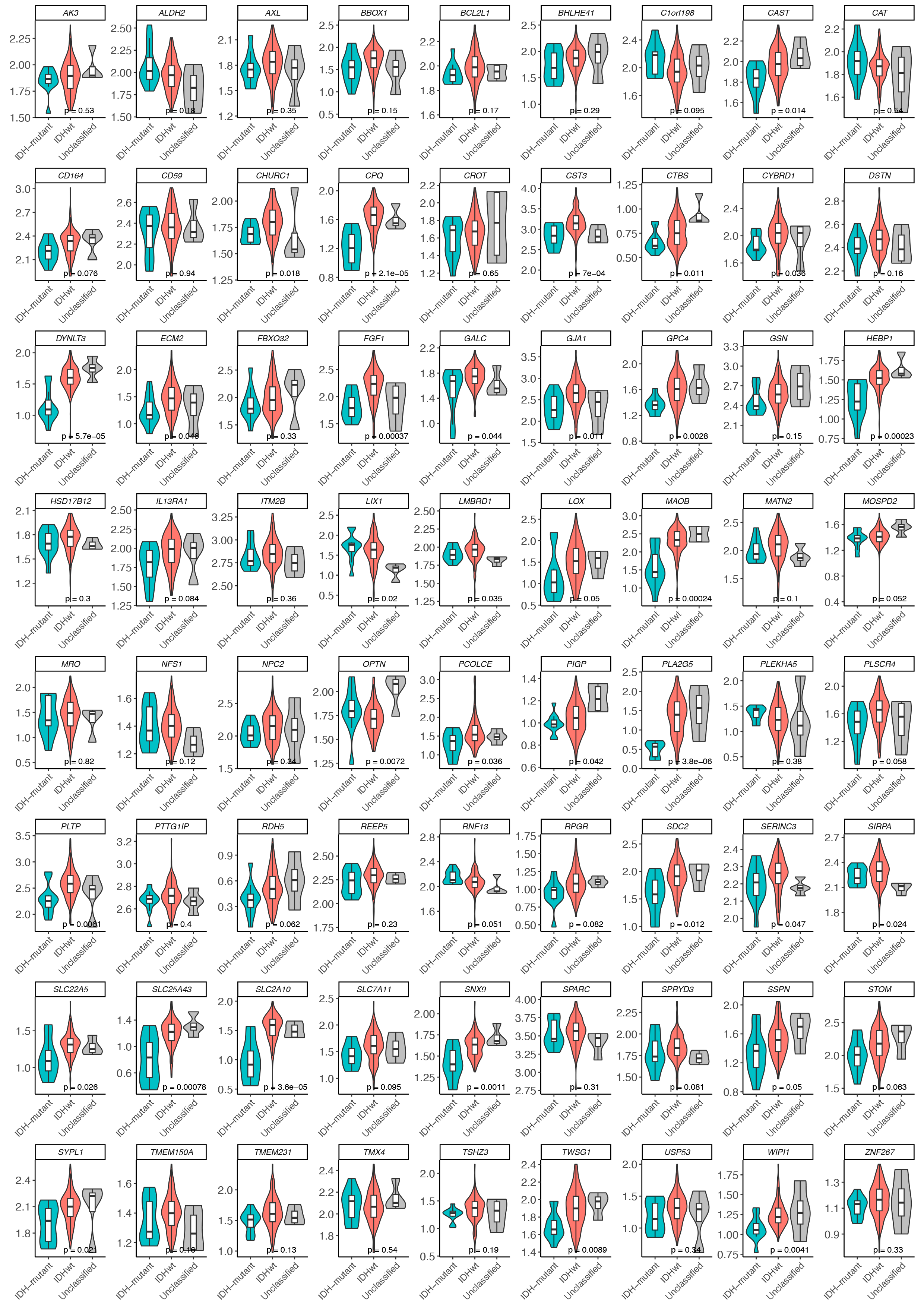

Supplement: Supplementary file 4 — Additional file 4: Figure S4. [file 12885_2024_11914_MOESM4_ESM.png]

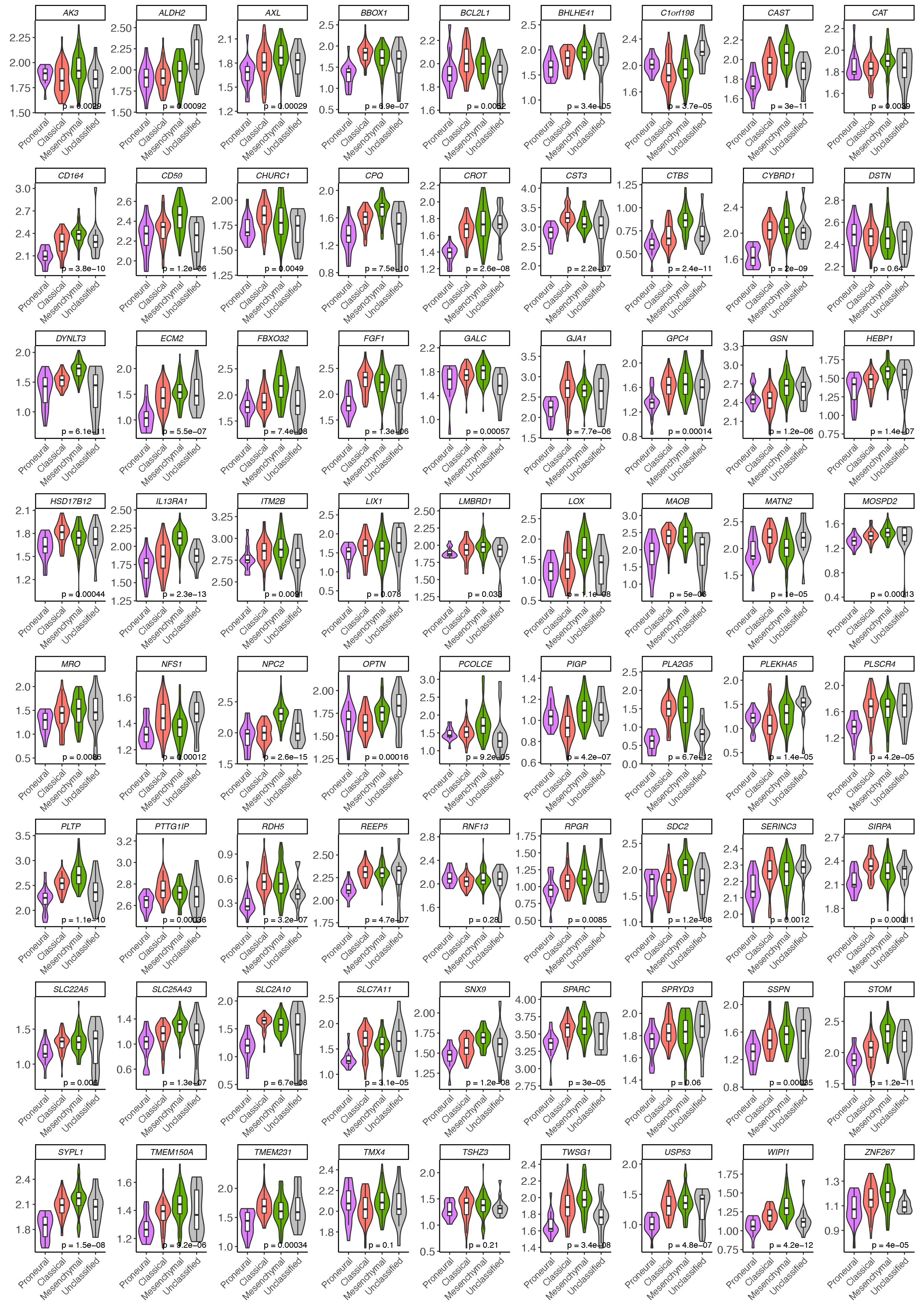

Supplement: Supplementary file 5 — Additional file 5: Figure S5. [file 12885_2024_11914_MOESM5_ESM.png]

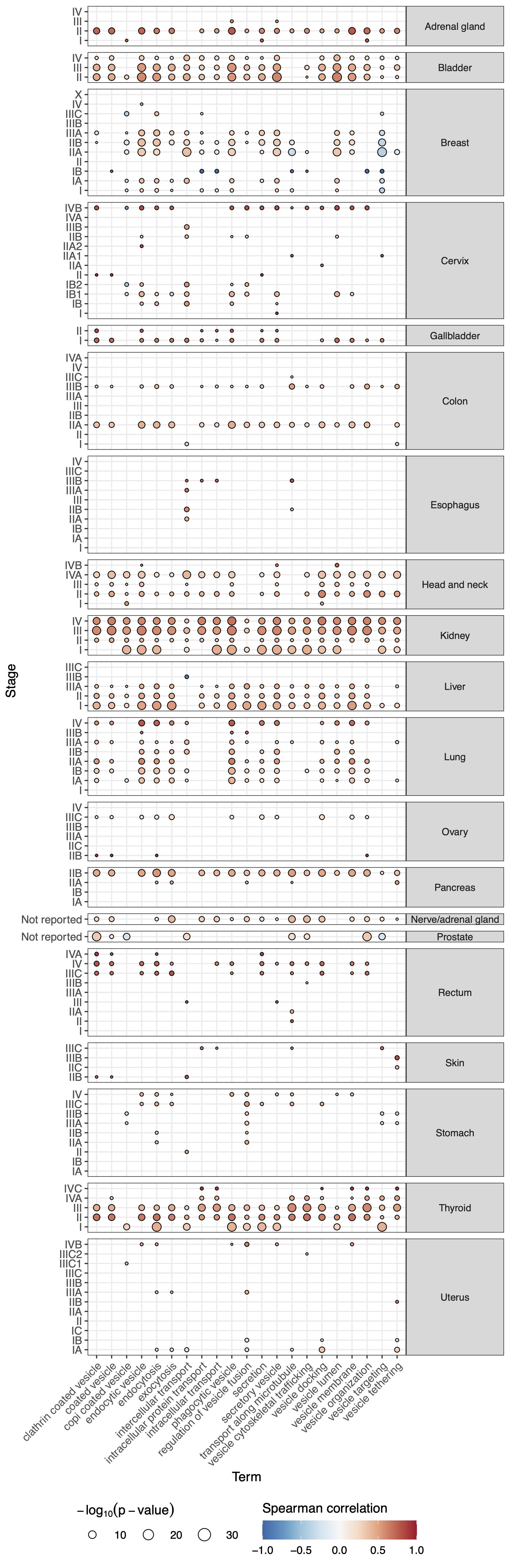

Supplement: Supplementary file 7 — Additional file 7: Figure S7. [file 12885_2024_11914_MOESM7_ESM.png]
